# Autophagy in *Plasmodium*, a multifunctional pathway?

**DOI:** 10.5936/csbj.201308002

**Published:** 2013-08-20

**Authors:** Adelaide U.P. Hain, Jürgen Bosch

**Affiliations:** a Department of Biochemistry and Molecular Biology, Bloomberg School of Public Health, Johns Hopkins Malaria Research Institute, Baltimore, MD 21205, United States

**Keywords:** *Plasmodium*, apicoplast, heme, Atg, digestive vacuole

## Abstract

Autophagy is a catabolic process that normally utilizes the lysosome. The far-reaching implications of this system in disease are being increasingly understood. Studying autophagy is complicated by its role in cell survival and programmed cell death and the involvement of the canonical marker of autophagy, Atg8/LC3, in numerous non-autophagic roles. The malaria parasite, *Plasmodium*, has conserved certain aspects of the autophagic machinery but for what purpose has long remained a mystery. Major advances have recently been gained and suggest a role for Atg8 in apicoplast maintenance, degradation of heme inside the food vacuole, and possibly trafficking of proteins or organelles outside the parasite membrane. Autophagy may also participate in programmed cell death under drug treatment or as a selective tool to limit parasite load. We review the current findings and discuss discrepancies in the field of autophagy in the *Plasmodium parasite*.

## Introduction

Classically, autophagy has been defined as a eukaryotic system for degradation of cellular material inside the lysosome. The best-studied form, macroautophagy, encapsulates the cytoplasmic material inside a double-membrane vesicle, the autophagosome that fuses with the lysosome. Once considered a nonselective bulk process, autophagy is now appreciated as a way to quickly respond to shifting cellular conditions and to selectively degrade damaged organelles, proteins, nucleic acids, and pathogens. Interest in autophagy has surged in the past five years and is now seen as a therapeutic target in many diseases including cancer, neurodegeneration, and infectious disease [1–3], where not just host autophagy but that of the eukaryotic pathogen plays a critical role in infection [4]. While much was known about the role of autophagy in the protozoans *Leishmania*, *Trypanosoma*, and *Toxoplasma* [5–7], the story was less conclusive for the malaria parasite *Plasmodium*, which kills nearly one million people each year [8], as well as the related species, *Theileria* and *Eimeria*. However, great advances have been made in the last year.

The first mention of plasmodial autophagy in the literature was in the 1970s by D.C. Warhurst and colleagues. They conducted extensive studies showing increased pigment clumping in “autophagic vacuoles” following chloroquine (CQ) treatment in blood stage parasites [9, 10]. In the 2000s, drug treatment was again reported to induce autophagic vacuoles and an autophagic death [11, 12]. However, Klionsky, a leading researcher in autophagy, has warned against the visualization of autophagosomes through electron microscopy as the sole evidence for an autophagic process [13]. The difficulty in culturing certain stages of the parasite, genetically manipulating the organism to make conditional knockouts, and to clone and express genes recombinantly all have contributed to a void of knowledge in this area. The last year witnessed a vast expansion in our understanding of autophagic pathways in the *Plasmodium* parasite.

## *Plasmodium* biology

The *Plasmodium* parasite has evolved distinctive biological mechanisms to invade and replicate within different cell types in both the mosquito vector and mammalian host as part of its life cycle. It is necessary to understand *Plasmodium* biology if one is to study autophagy in the context of this unique unicellular eukaryote. During a mosquito blood meal, elongated sporozoites are injected into the host and invade liver cells. Within a hepatocyte the sporozoite transforms into a round trophozoite and organelles used for the invasion process are expelled [14, 15]. The parasite uses a form of asexual replication, schizogony, to produce hundreds to thousands of teardrop-shaped merozoites. Extensive remodeling of the host cell creates a merosome, which contains the merozoites that are released into the bloodstream to continue the next stage of infection, the symptomatic erythrocytic stage [16, 17]. A merozoite invades a red blood cell (RBC), develops into the ring stage form, converts into a trophozoite and then undergoes schizogony to produce more merozoites for sequential rounds of invasion and replication. During the trophic stage, hemoglobin is taken up from the host RBC cytoplasm and transported to the food vacuole (FV), a lysosome-like compartment where it is degraded to provide amino acids for parasite growth. Some merozoites are shunted from this cycle to produce gametocytes, which are subsequently taken up by a mosquito during a blood meal, continuing the *Plasmodium* life cycle.

### Overview of Macroautophagy

**Figure 1:**
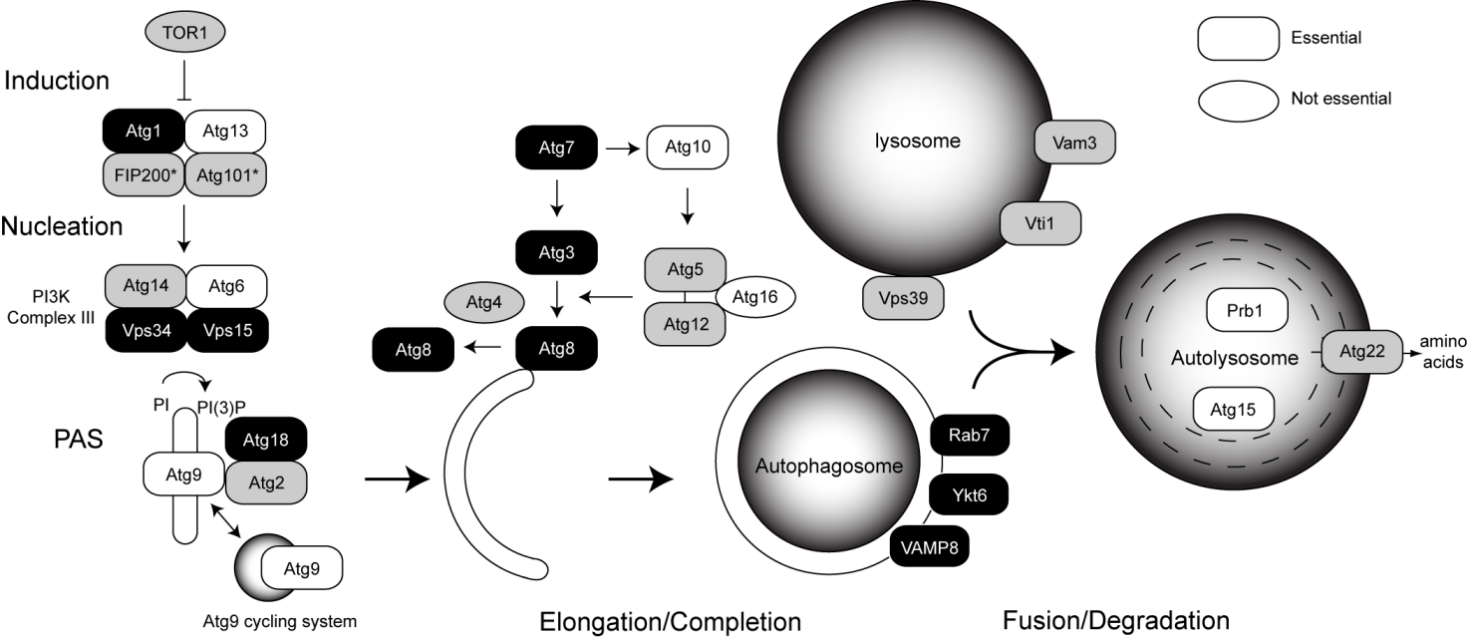
Conservation of autophagy proteins in *Plasmodium*. Proteins essential for macroautophagy are depicted as rectangles. Ovals represent proteins whose essentiality is not known. Black indicates strong evidence for homology (e-value less than 1e-10 in initial BLAST within PlasmoDB and reciprocal identification using PSI-BLAST with e-value less than 1e-20). Gray indicates homology less clear (initial e-value cutoff of 0.012 and identification of initial query in either PSI- or DELTA-BLAST with e-value that may not be significant). White indicates no orthologue was identified. *Human proteins FIP200 and Atg101 thought to be functional homologues of yeast Atg31/Atg17Atg29.

Although over 30 AuTophaGy (Atg) proteins have been identified in yeast, not all are essential to macroautophagy (Figure 1). Autophagy induction in yeast (and mammals) begins with the Atg1 (ULK1 in mammals) protein kinase complex, which is inhibited by the target of rapamycin, TORC1 (mTOR in mammals) complex under homeostasis. Nutrient deprivation or rapamycin treatment alleviates the negative regulation on the Atg1 (ULK1) complex [18]. Atg1 and Atg13 form a complex with Atg31/Atg17/Atg29/Atg11 in yeast and FIP200/Atg101 in mammals at the pre-autophagosomal structure (PAS) [19, 20]. Atg17 has been implicated as the most upstream scaffold for protein recruitment to the PAS [21]. Phosphorylation of substrates by Atg1 (ULK1) activates the Phosphatidyl-Inositol 3-phosphate Kinase (PI3K) class III complex, composed of Vps34, Atg14, Vps15/p150, and Atg6 (Beclin1 in mammals) which converts phosphatidylinositol (PI) into phosphatidylinositol triphosphate (PI(3)P) at the site of the PAS [22]. This creates a binding site for Atg18 (WIPIs in mammals) and Atg2 at the isolation membrane, also requiring association with the transmembrane protein Atg9 [23]. Elongation of the membrane and closure of the autophagosome requires covalent attachment of the C-terminal glycine of Atg8 (LC3 in mammals) to phosphatidylethanolamine (PE) in the phagophore membrane [24]. Atg8 is a ubiquitin-like protein (Ubl) that requires the protease Atg4, an E1-activating enzyme, Atg7, and an E2-conjugating enzyme, Atg3 for lipidation [25, 26]. Another autophagy Ubl protein, Atg12, is covalently attached to Atg5, requiring Atg7 as well, and its own E2, Atg10 [27]. The Atg12-Atg5 conjugate forms a complex with Atg16 to drastically increase conjugation of Atg8, presumably by forming a scaffold with Atg3 at the membrane [28]. The autophagosome seals and fuses with endocytic compartments and the lysosome, where the material is broken down and recycled back to the cytoplasm.\

To obtain an overview of the system, we performed bioinformatics analysis to identify putative orthologues of autophagy proteins in *Plasmodium falciparum*. While such lists have been published, our analysis focused on the minimal machinery needed for macroautophagy in yeast [4, 6, 29–31]. The coordination of events in macroautophagy is diagrammed in Figure 1, with putative homologues identified in *Plasmodium* colored in black and less reliable identifications in gray. Although several hits were identified for the nutrient sensor TOR, these hits have clearer homology to Phosphatidyl-Inositol 4-Kinases. Of the proteins involved in autophagy induction, Atg1 and its mammalian binding partners, FIP200 and Atg101 appear to have orthologues in *Plasmodium*, while Atg13 appears absent. The C-terminal Atg13-binding domain is also missing from PfAtg1. It contains several potential Atg8-interacting motifs, which mediate Atg1 binding to Atg8 in an Atg13-independent manner in yeast and humans [32]. PfVps34 exists in *P. falciparum* and is one of the better-characterized proteins. Vps15 and Atg14, also members of the PI3K complex were identified, but Atg6 was not.

Atg9 is an essential transmembrane protein for autophagy thought to supply Golgi-derived membranes to the PAS during initiation [33, 34]. None of the significant BLAST hits for Atg9 are predicted to contain any of its six transmembrane domains. Atg18 likely has a homologue in *Plasmodium* and contains the FRRG motif necessary for binding to PI(3)P and Atg18-Atg2 recruitment to the PAS as well as WD40 repeats for beta propeller formation. Atg18 is thought to aid recruitment of Atg8 to the PAS and protect it from premature Atg4 cleavage [35]. A putative homologue for Atg18's binding partner, Atg2, was also identified but is annotated as a putative rhoptry protein.

Atg8, Atg7, and Atg3 orthologues are present. Atg8 contains a C-terminal glycine in *Plasmodium* [36]. Similar Atg8 homologs have been identified in plants and other protists including *Toxoplama* [37, 38]. Therefore, the putative Atg4 orthologue is likely important for recycling improperly conjugated Atg8 [39]. Putative homologues for the Ubl, Atg12 and its target, Atg5, were identified. However, the C-terminal glycine of PfAtg12 and its E2 enzyme needed for covalent attachment are absent. It is unclear if Atg16, which forms a 350 kDa complex with Atg12-Atg5 is present. It is essential *in vivo* but not *in vitro* for Atg8 lipidation [28, 40].

Fusion of both autophagosomes and endosomes with the lysosome requires Rab7 and various SNAREs [41, 42]. Apicomplexans contain a basic set of Rabs, including Rab7 [43]. Putative homologues for essential SNARES including Vti1b and VAMP8 also exist in *Plasmodium*. The lipase for breaking down the autophagosome membrane, Atg15, and the vacuolar proteinase Prb1 are absent [44, 45]. However, the parasite contains numerous falcipains in the food vacuole for degrading hemoglobin. The lysosomal amino acid efflux pump Atg22 is present [46]. More information, including the identity of hits is in supplementary table 1 (ST1).

## Methods

The genome sequence of *P. falciparum* was published in 2002 and is stored along with various data associated with each gene in the *Plasmodium* Genome database (PlasmoDB) [47, 48]. The amino acid coding sequence of *Saccharomyces cerevisiae* and *Homo sapiens* ATG genes were queried for putative homologues using the WU-BLAST program within PlasmoDB. Hits were identified based on the Expect (e)-value of the BLAST search, as well as annotation in PlasmoDB, any functional knowledge, conservation among all the plasmodial species sequenced, and the length of the hit in comparison to the query. Of note, the most likely homologue did not necessarily have the highest e-value in the initial PlasmoDB BLAST search. Additionally the species used as the query had a major impact on the returned BLAST hits. Often the same hit was identified with both the human and yeast homologue query but sometimes a strong hit was only identified with one species. In a few cases other species were used as a query (ST1). An inverse BLAST was performed with the potential hits using NCBI Position-Specific Iterated (PSI) BLAST against the *S. cerevisiae* or *H. sapiens* non-redundant protein sequence database, and if that failed, with the Domain Enhanced Lookup Time Accelerated (DELTA) BLAST. Hits were additionally screened in Pfam to see if they contained the domains or motifs important to that homologue's function, such as coiled-coil domains and lipid binding motifs. For transmembrane proteins, the TMHMM server 2.0 (http://www.cbs.dtu.dk/services/TMHMM/) was used to compare predicted membrane topology.

### Localization of the autophagy marker, Atg8

Atg8/LC3 is the classical autophagy marker as it is covalently attached to the pre-autophagosome membrane at an early stage and stays conjugated to the autophagosome during fusion with the lysosome. In yeast and mammals, Atg8/LC3 has a diffuse cytoplasmic distribution under nutrient rich conditions that becomes punctate upon autophagy induction [25]. The lipidated fraction of Atg8 can also be visualized as a faster migrating band with high percentage or urea-contaning gels in SDS-PAGE [26].

Several groups have examined the localization of Atg8 in different *Plasmodium* species and stages. Under normal conditions, *P. berghei* liver and *P. falciparum* blood stage Atg8 are in punctae that are nearby and partially colocalize with the apicoplast marker, acyl-carrier protein (ACP) [30, 31, 49]. All members of the phylum Apicomplexa, with the exception of *Cryptospiridium*, contain apicoplasts, plastid organelles analogous to chloroplasts in plants, acquired through endosymbiosis. The organelle is essential to *Plasmodium* survival in both asexual stages and is the site for synthesis of iron-sulfur clusters, isoprenoids, and fatty acids [50]. Localization to other organelles, such as mitochondria, rhoptries, micronemes, and dense granules was negative in the schizont, while transgenic Atg8 lacking the C-terminal glycine for lipidation showed diffuse localization, validating the results of these studies [30]. In hepatocytes, PbAtg8 localization along the apicoplast was observed 14 hours post-invasion and increased with organelle size throughout the liver stage [49].

Roepe and Sinai also observe punctate PfAtg8 in blood stage trophozoites, but co-localization with ACP, or other organelle markers, was not assessed [5]. Interestingly, when cells were transferred to media lacking amino acids and glucose for 6 hours, PfAtg8 redistributes to a wider punctate staining that seems to be inside the host red blood cell cytoplasm. Similar re-localization was seen in treatment with high levels of CQ [5]. Langsley and colleagues also starved blood stage parasites in media lacking amino acids. At 4 hours, they observed localization of PfAtg8 to double-membrane structures also containing PfRab7 that were near or inside the food vacuole, visualized with electron micrographs, implicating the food vacuole as the final digestive compartment in a *Plasmodium* autophagic-like process [51].

PfAtg8 shows elongated tubular localization in oocytes obtained from mosquito midgut and sporozoites from the mosquito salivary glands, reminiscent of apicoplast morphology under normal cell conditions [49].

### Expression data for autophagy genes

**Figure 2:**
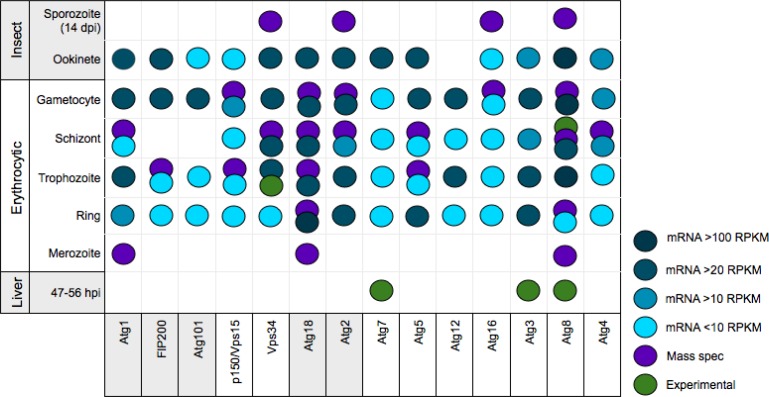
Compiled expression data of autophagy proteins during *Plasmodium* life cycle. RNA-Seq and mass spectrometry data from PlasmoDB and data from published studies is compiled. All data is for *P. falciparum* homologues (ST1), except liver stage data from *P. berghei*.

PlasmoDB houses RNA-Seq data during the blood stage for each gene [52]. Additionally, several widespread mass spectrometry studies have provided data on protein expression in the liver and blood stages [53–58]. There is also specific transcript, immunoblotting and/or immunofluorescence data for a small subset of autophagy genes [30, 31, 49, 59]. Evidence for expression from these various sources is compiled in Figure 2 and supports an important role for autophagy proteins during the late blood stage, where both hemoglobin digestion and apicoplast growth is maximal. Less data is available for non-blood stages. Atg8, Atg3, and Atg7 are transcribed during all life cycles of *P. berghei* [49].

## Potential Functions

**Figure 3:**
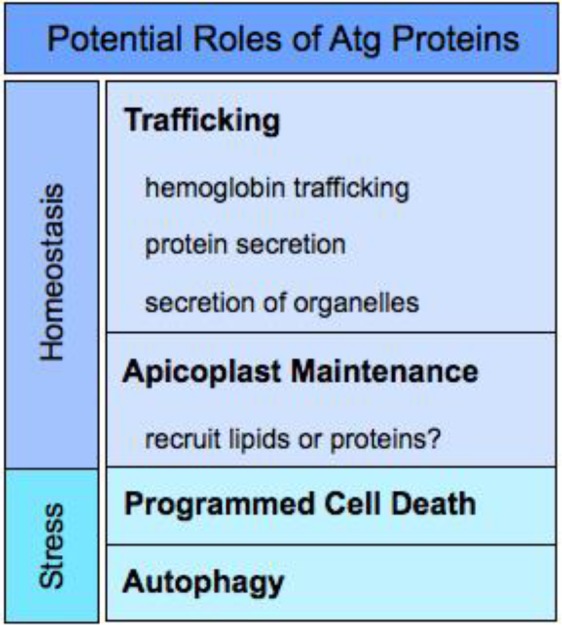
Potential Roles for Atg proteins in *Plasmodium*.

Strictly speaking, confirmation of macroautophagy in *Plasmodium* would require 1. the presence of Atg8-labelled double-membrane vesicles, 2. fusion of these vesicles with a lysosomal-like compartment, and 3. breakdown and recycling of the autophagosome cargo. Recently, Atg8 has been observed on double-membrane vesicles near and inside the food vacuole, an acidic compartment that most likely serves as a lysosome in the parasite, providing intriguing evidence for macroautophagy [51]. Additionally, there is evidence of autophagic-like and distinct functions utilizing the autophagic machinery. The unique biology of the parasite as well as the adaptability of the machinery in other organisms for non-autophagic purposes makes alternative roles quite feasible and even likely [60]. Atg8's essentiality in *Plasmodium* is intriguing, as Atg8 knockouts are viable in yeast, which suggests an alternative or additional vital function(s) in the parasite [24]. In the highly related Apicomplexa, *Toxoplasma*, the E2 conjugating enzyme for TgAtg8, TgAtg3, is also essential [38]. Here we will present current thinking for the role of Atg8 and the autophagy machinery in *Plasmodium*, which is summarized in Figure 3.

## Vesicular Trafficking

### Protein Secretion

The *Plasmodium* parasite initiates extensive remodeling of the host cell upon infection by exporting its own proteins into the host cell. *Plasmodium* is encapsulated by a parasitopherous vacuole within host cells. Proteins targeted for export to the host cytosol or plasma membrane must be trafficked out of the parasite plasma membrane and the PV, necessitating novel pathways for protein secretion. It is possible that exophagy, where proteins inside autophagosomes are re-routed to the plasma membrane is involved [61]. Exophagy of Acb1 in yeast utilizes the autophagosomal machinery as well as GRASP65 and the t-SNARE, Sso1, which have putative homologues in *Plasmodium* [62, 63]. In the current model, autophagosomes fuse with multivesicular bodies to form amphisomes. Exophagy utilizes the COPII machinery but bypasses Golgi processing and is packaged into autophagosomes that fuse with plasma membrane [64].

A recent review suggests that vesicles originating at the ER via the formation of PI(3)P are destined for export to the host cytoplasm [65]. Vps34, also known as type III PI3K, is a lipid kinase at the ER that produces PI(3)P on the outer leaflet, important for recruitment of proteins to the phagophore and proper curvature of the autophagosome. PfVps34 purified from parasite lysate has kinase activity that can be inhibited by the well-characterized class III PI3K complex inhibitor, wortmannin. Endogenous PfVps34 localizes to the food vacuole and vesicular structures near the host plasma membrane in the blood stage, partially co-localizing with the VARC protein, a virulence factor in *P. falciparum* that is exported to the erythrocyte surface [59]. One of the more characterized routes of protein export beyond the PV utilizes a PEXEL motif for recognition. Recently, PI(3)P was shown to bind the PEXEL motif at PI(3)P-enriched regions in the ER [66].

We previously identified the small GTPase sar1 in a pull-down with tagged PfAtg8 and blood stage *P. falciparum* lysate [36]. Sar1 is a component of the COPII vesicle machinery for trafficking between the ER and Golgi. Sar1 was shown to have similar localization to the ER and small vesicle compartments in *P. falciparum* blood stages as in yeast, at the ER site for secretion of proteins [67, 68]. The role of other Atg proteins in protein secretion is not known. As will be discussed below, PfAtg8 localization in the RBC cytoplasm is reported, but only under non-homeostatic conditions and co-localization with VARC or the Maurer's cleft, an intermediate compartment for protein export in the RBC cytoplasm, was not examined [5].

### Organelle expulsion

The *Plasmodium* parasite undergoes major morphological changes in the early liver stage, converting from a motile, slender sporozoite into a round trophozoite. During this transformation, organelles used for liver cell invasion, including the inner membrane complex, rhoptries, and micronemes, are expelled from the parasites [14]. Treatment of *Plasmodium* sporozoites with 3-methyladenine (3-MA), which targets Vps34, needed for PI(3)P production and phagophore formation, delays conversion into trophozoites [69]. Electron micrographs of liver stage parasites revealed double-membraned structures morphologically similar to autophagosomes containing micronemes leading to the hypothesis that exophagy of organelles could be important for differentiation in the liver stage in the absence of an extensive intracellular degradative system [6].

### Trafficking to food vacuole

Chloroquine (CQ) is a lysosomatropic agent that prevents autophagosome-lysosome fusion [70, 71]. In other studied Eukarya, CQ causes an accumulation of Atg8 lipidated to autophagosomes in starvation and non-starvation conditions [71]. A longstanding antimalarial, CQ accumulates in the food vacuole and prevents hemozoin dimerization and hemoglobin digestion [72], starving the parasite of amino acids. Varying responses are observed in *Plasmodium* upon CQ treatment and this appears to be due in part to the concentration that is used and the duration studied. In CQ sensitive strains, such as 3D7, ten to 100 nM is cytostatic whereas micromolar amounts are cytocidal [73].

In early to mid-trophozoite stages, host cytoplasm is endocytosed, delivering hemoglobin to the food vacuole as a source for amino acids. It has long been reported that CQ treatment led to production of vesicles inside the *Plasmodium* parasite [10, 11], which were assumed to be autophagic vesicles. Extended incubation (12 hours) with 120 nM CQ leads to a build-up of hemoglobin-containing vesicles, likely by preventing fusion of these vesicles with the food vacuole, preventing hemoglobin digestion and starving the parasite of amino acids [74]. Sinai and Roepe report drastic re-localization of PfAtg8 from the parasite to the RBC host cytoplasm with either prolonged starvation or high cytocidal concentrations of CQ [5]. The authors hypothesize that PfAtg8 may be involved in vesicular trafficking of hemoglobin or other nutrients under starvation conditions.

In contrast, Kitamura et *al*. do not see a change in PfAtg8 localization or protein levels in response to CQ treatment as would be expected if Atg8-containing vesicles could not fuse with the food vacuole (FV) or if Atg8 was exported into the RBC cytoplasm. They used 100 and 300 nM concentrations of CQ, which are cytostatic, not cytocidal, and may not be sufficient to induce a lysosomatrophic effect, as 100 µM is typically used in mammalian cell lines to block autophagy [73]. The treatment period in the paper was also shorter than with Sinai and Roepe.

During the preparation of this review it was shown with immuno-EM that endogenous PfAtg8 was located at Rab7-containing vesicles near and inside the food vacuole in parasites starved of amino acids for 4 hours. The authors never saw PfAtg8 in the host cytoplasm [51]. Immunofluorescence, co-staining for anti-hemoglobin antiserum and anti-Atg8 antibodies, would elucidate whether PfAtg8 is involved in trafficking of hemoglobin. Due to the differences in *P. falciparum* strain sensitivity to CQ and different effects observed with varying concentrations and exposure times, amino acid deprivation, the canonical inducer of autophagy, is a more suitable tool to study this pathway in *Plasmodium*.

Additional support for autophagic functions in hemoglobin degradation is garnered by localization of PfVps34 to the food vacuole as well as vesicles in the host cytoplasm [59]. Strikingly, the PI3K inhibitor, wortmannin (10 µM, 5 hour treatment), caused accumulation of hemoglobin-containing endocytic vesicles within the parasite and subsequently less in the FV, indicating a defect in digestion concomitant with decreased parasite growth. They suggest the class III PI3K complex is important for hemoglobin endocytosis and trafficking to the FV in *Plasmodium*. While the effect of wortmannin on *Plasmodium* Atg8 was examined under normal conditions, it was not tested under starvation conditions to see if treatment prevented PfAtg8 localization to vesicles and the FV. However another PI3K inhibitor, 3-MA, inhibited re-localization of PfAtg8 to the host cytoplasm upon starvation or CQ treatment, implicating involvement of Atg8 in trafficking of theses hemoglobin-containing vesicles [75].

The malaria parasite does not obtain isoleucine from hemoglobin and is dependent on the host for this amino acid. *Plasmodium* can survive up to 72 hours without exogenous isoleucine, suggesting a survival mechanism is present. Since digestive vacuole proteases are essential to survival, an autophagic mechanism was suggested [76]. As mentioned previously, amino acid starvation induces a re-localization of Atg8 to either the periphery of the parasite or to vesicles near or within the food vacuole depending on the experimental conditions [5, 51]. It would be of great interest to determine if isoleucine deprivation alone induced co-localization between PfAtg8 and PfRab7 at the food vacuole.

The product of PI3K, PI(3)P, is enriched at both the apicoplast and the food vacuole and one of the few PI(3)P-binding proteins identified in *Plasmodium*, FCP protein, is trafficked to the FV using an unknown mechanism, which some suggest may be similar to the cytoplasm-to-vacuole targeting pathway in yeast [77]. However PfAtg8 re-localization away from the apicoplast appears to require a signal such as drug treatment or starvation and physiological relevance of these conditions is unclear. One could speculate a role for detoxification of drugs or increased uptake of nutrients through exophagy of importer proteins.

## Programmed Cell Death

Programmed cell death (PCD) pathways are increasingly appreciated as an important way to modulate host-pathogen responses and limit parasite load [78]. Autophagy is a recognized mechanism of cell death in other eukarya where the balance between pro-survival and pro-death is tightly regulated [79]. Autophagic cell death has previously been reported for the protozoan parasites *Toxoplasma* and trypanosomes and less conclusively in *Leishmania*, but there is controversy as to the legitimacy of a regulated rather than incidental mechanism of cell death in protozoa [7, 80–83].

Treatment of *P. falciparum* erythrocytes with chloroquine (180 nM), SNAP, a nitric oxide donor, and staurosporine, an apoptosis inducing agent, led to cell death that was described as autophagic-like because of distinct vacuolarization of the parasite cytoplasm and presence of double-membrane structures, whereas the nuclear morphology remained in tact and other apoptotic markers were absent [11]. The morphology of this cell death is similar to that observed in retinal pigment epithelial cells upon treatment with much higher concentrations (120 µM) of CQ and is thought to be the mechanism for retinotoxicity associated with CQ. In this case, they also did not see hallmarks of apoptosis but did see increased lipidation of LC3 to autophagic vacuoles (AVs) and a block in degradation of the autophagy-specific substrate, p62 [84]. Autophagic vacuoles were also observed in *P. berghei* blood stage parasites dying after treatment with dihydroartemisinin [12]. In this study, the authors concluded, “Since the food vacuole membranes were affected, the nutrition of parasites was blocked and the autophagic vacuoles formed quickly as a result of amino-acid hunger of parasites.” However distribution of Atg8 was not investigated.

Recently, it has been reported that after invasion of hepatocytes, a significant fraction of *P. berghei* parasites die through an autophagic mechanism [30]. This was observed in cell culture and in mice, indicating it is a physiological mechanism. The process is restricted to the later stages of development. Eickel et *al*. observed double membrane structures that appeared similar to those reported by Meis and Verhave in 1985, then described as “membranous whorls” [15]. Yet, fluorescence microscopy revealed that PbAtg8 never localized to these vesicular structures and remained associated with the apicoplast, determined by colocalization with ACP, and its organellar fragments throughout the cell death event [30]. Treatment with rapamycin (500 nM), which potently induces autophagy in yeast through inhibition of TOR, decreased parasite size and changed apicoplast morphology, but did not affect PbAtg8 localization. However, there is no known TOR orthologue in *Plasmodium*, and the observed effect on parasite growth could be mediated through off-target effects or effects on host autophagy [85]. The authors speculate that autophagy occurs in the parasite but is independent of Atg8. Although Atg8 is the canonical autophagic marker, there are reports of macroautophagy that is independent of LC3 conjugation [86]. However this alternative pathway requires ULK1, Rab9, and Beclin 1 and Rab9 and Beclin 1 orthologues have not been identified in *Plasmodium*.

## Apicoplast Maintenance

The apicoplast is vital to survival in both mammalian stages. A gradual increase in apicoplast size begins in the trophozoite stage. Dramatic elongation and branching of the organelle occurs in the late trophozoite/early schizont stage. Division of the apicoplast occurs late in the schizont and ultimately results in each merozoite containing one apicoplast [87]. If the autophagic machinery delivers lipids or proteins to the growing apicoplast membrane, expression of its component proteins is necessary by the trophozoite stage. As shown in Figure 2, this appears to be the case.

Pf/PbAtg8 localizes to the apicoplast in liver and blood stages under normal conditions. Electron micrographs and biochemical data indicate that Atg8 is conjugated to the outermost membrane of the apicoplast [31]. *Plasmodium* treated with chloramphenicol leads to loss of the apicoplast, which is normally lethal, but supplementation with isopentenylpyrophosphate allows the parasite to survive during the RBC stage [88]. Tomlins et *al*. observed PfAtg8 localization under these conditions. Strikingly, PfAtg8 was located at vesicular structures that also contained the apicoplast-targeted protein LipDH1, suggesting a role in trafficking [51].

Treatment of parasites with the Vps34 inhibitor, wortmannin (10 µM), did not change the localization of PfAtg8 at the apicoplast in the blood or liver stage suggesting Atg8 localization to the apicoplast is constitutive and not induced by the class III PI3K complex [31, 51]. However PfVps34 is also located at the apicoplast and its product, PI(3)P, is enriched at the apicoplast and nearby single-membrane vesicles, in addition to the food vacuole [89]. PI(3)P production is maximal in later blood stages and Tawk et *al*. suggest that PI(3)P vesicles transport lipids or proteins to the apicoplast, as is the case in *T. gondii* [90]. In agreement with this vital role, PI3K is essential in *P. berghei* [89]. As of now, there are no studies describing co-localization between PI3K and Atg8.

Interestingly, although PbAtg8 localized to the apicoplast in the liver stage, its E1 activating and E2 conjugating enzyme, Atg7 and Atg3, were located at the mitochondria, leading the authors to question whether they interact with PbAtg8 [30]. However, localization was only described at 52 h.p.i., at which point PbAtg8 is already lipidated to the outer membrane of the apicoplast [49]. It is difficult to imagine how Atg8 is lipidated without an activating and conjugating enzyme, other than the possibility that an E1 and E2 enzyme function has divergently evolved. Further localization studies in other stages and time points, as well as knockout of Atg3 and Atg7 would address these questions.

## Current Model

During normal cellular conditions, the autophagy machinery, including Atg8 and a pool of Vps34 is localized to the apicoplast where it traffics proteins and/or lipids to the apicoplast and later recruits proteins/lipids for increased growth in preparation for cellular division. This does not require induction and occurs both in the liver and blood stage and most likely in the oocyst and sporozoite mosquito stages as well. This role most clearly explains PfAtg8 and Vps34's essential phenotype as autophagy would only be required under starvation conditions and protein trafficking to the membrane is most likely not essential, especially in *in vitro* conditions. During cases of extreme duress, such as starvation or drug treatment, the parasite induces an autophagic-like response that increases endocytosis of host cytoplasm and digestion in the food vacuole. If the stress conditions persist and the parasite cannot cope, the balance may be tipped to autophagic death. This may also be a mechanism to limit the number of parasites in the liver stage such that only the most robust parasites continue to the blood stage of infection. What little evidence exists suggests this cell death is independent of Atg8, but this remains to be further clarified [30].

## Targeting Autophagy

It appears that Atg8, due to its unique ability to be covalently attached to membranes and to bind diverse proteins through adaptors has been conserved throughout Eukarya, even when other autophagy proteins are not. The list of non-autophagic functions for Atg8 homologues continues to grow and includes functions independent of membrane conjugation [91, 92]. Since *Plasmodium* Atg8 knockouts are nonviable and conditional knockouts are difficult in *Plasmodium*, a selective inhibitor preventing lipidation of Atg8 would be an invaluable tool that could also serve as a potential antimalarial therapeutic. Plasmodial Atg8's essentiality and our recently elucidated crystal structure make PfAtg8 a prime target for structure-guided drug design [36]. It would be of great interest to determine whether apicoplast morphology changes upon Atg8 inhibition, particularly during schizogony when it grows in size. Additionally, would parasites exhibit similar cell death by CQ when Atg8 is inhibited? Or conversely, would they be more susceptible?

While two independent studies concluded that PbAtg8 could not complement an Atg8 knockout in yeast [30, 49] a recent study showed PfAtg8 to be a functional homologue for ScAtg8 [30, 49, 51]. This discrepancy between the species is unexpected as *P. berghei* and *P. falciparum* Atg8 are 88% identical to each other and 40% identical to yeast Atg8. Transgenic PfAtg8 was not lipidated in mammalian cells, indicating PfAtg8 cannot complement the human homologue and PfAtg3 could not interact with human LC3 [36, 49]. We demonstrated that although PfAtg3 utilizes an Atg8-interacting motif similar to the human system, it also interacts through a short loop in PfAtg8 not present in humans [36]. Likely, other autophagy protein-protein interactions have similar divergences in their interaction surface and represent potential drug targets. The PfVps34 C-terminal region has 40% similarity and 23% identity to the human homologue but contains a substantial 1000 amino acid N-terminal extension with no known domain structure in Pfam. Perhaps its function at the apicoplast is mediated through this region? Unique insertions like these would serve as a platform for novel drug design against autophagic and trafficking pathways that increasingly seem important for multiple stages of the *Plasmodium* life cycle. However, their function needs to be elucidated.

## Concluding Remarks

Remarkable progress has been made in the last year, dissecting the role of the autophagic machinery in *Plasmodium*, but many questions remain. The field is in agreement that Atg8 is located at the apicoplast, but it is not known if Atg8 is essential for the integrity of the organelle. Does Atg8 transport proteins, phospholipids, or both to the apicoplast and how is it targeted?

Starvation or CQ treatment leads to re-localization of Atg8 away from the apicoplast and is suggestive of a pro-survival mechanism under stress. But does this represent a typical autophagy pathway whereby parasitic proteins or organelles are recycled? The presence of Atg8 in the host cytoplasm suggests Atg8 is involved in trafficking that could include parasite protein export, host protein or heme import or even an attempt at detoxification of drugs such as CQ.

What, if anything, acts upstream of Atg1 to induce autophagy under starvation conditions? In yeast, cAMP-dependent protein kinase (PKA) signaling regulates Atg1 independent from TOR and may be a regulator in *Plasmodium* [93]. PfVps34 is phosphorylated in the parasite at a predicted PKA site [94]. PfeIK1, an orthologue for eukaryotic initiation factor 2α kinase, has been implicated in the amino acid starvation response in *Plasmodium* and has been suggested as a possible mediator of autophagy induction [5, 95]. At this time, there are more questions than answers, but it is encouraging to see that the field embraces autophagy in apicomplexa as a highly interesting research area. It can be expected that progress towards intervening molecules will be made and these will further facilitate biological research questions.

## Supplementary Material

Autophagy in *Plasmodium*, a multifunctional pathway?
